# Combination Image-Guided and Antibody-Targeted α-Therapy Before Targeted Immunotherapy for Treatment of Solid Tumors

**DOI:** 10.2967/jnumed.125.270266

**Published:** 2025-10

**Authors:** Maciej Kujawski, Eric Aniogo, Patty Wong, Susanta Hui, Hemendra Ghimire, Erasmus K. Poku, Paul J. Yazaki, Jeffrey Y.C. Wong, John E. Shively

**Affiliations:** 1 Department of Immunology and Theranostics, Beckman Research Institute, City of Hope, Duarte, California;; 2 Department of Radiation Oncology, Beckman Research Institute and Medical Center, City of Hope, Duarte, California; and; 3 Department of Radiopharmacy, Beckman Research Institute, City of Hope, Duarte, California

**Keywords:** image-guided radiotherapy, targeted α-therapy, targeted immunotherapy, radioimmunoimaging, radionuclide therapy

## Abstract

The rationale of this study was to evaluate the efficacy of 3 types of targeted therapy for solid tumors, comprising image-guided radiation therapy (IGRT), low-dose targeted α-therapy (TAT), and antibody-targeted interleukin-2 immunocytokine therapy, with the expectation that the combination of 2 types of targeted radiation therapy would perform better than either monotherapy with immunocytokine therapy. **Methods:** Carcinoembryonic antigen (CEA)–positive syngeneic breast and colon tumors in CEA transgenic animals were treated with single-dose IGRT (10 Gy) and 2 different regimens of fractionated IGRT (4 doses of 2.5 Gy or 4 doses of 3.65 Gy), with and without low-dose TAT (37 kBq of ^225^Ac-DOTA-anti-CEA) to optimize doses and tumor models for the combination of 2 types of IGRT plus TAT with immunocytokine therapy. **Results:** In preliminary PET imaging in the breast cancer model, fractionated IGRT (4 doses of 2.5 Gy) provided better antibody tumor penetration than did single-dose IGRT (10 Gy). Similarly, tumor regression and survival were superior with IGRT when combined with low-dose TAT, followed with best rechallenge responses in the groups treated with 4 doses of 2.5 Gy combined with TAT. Since comparable results were obtained in the colon cancer model, triple therapy (fractionated IGRT plus low-dose TAT followed by immunocytokine therapy) was studied in the colon cancer model, demonstrating complete cures in the majority of mice and rejection of all rechallenges. When the study was repeated for immunophenotyping 2 d after the completion of therapy, significant increases in natural killer and CD8-positive/interferon-gamma–positive cells were observed with triple therapy. Moreover, the changes in the tumor microenvironment, as reflected by the reduction of macrophages and infiltration of granulocytes and monocytes, was an important feature of these therapies. The memory phenotypes of the CD8-positive cells in tumor-draining lymph nodes and tumors showed significant increases of T cells with central memory versus naïve phenotypes in untreated controls. Interestingly, in contrast to the spleens and tumor-draining lymph nodes, there was almost a complete lack of naïve CD4-positive cells in the control tumors, a situation that was reversed by all 3 types of therapy, with the combination of IGRT, TAT, and immunocytokine therapy exhibiting the highest increase. **Conclusion:** Triple targeted therapy had the best therapeutic effects in a solid tumor model as evidenced by tumor cures, rejection of tumor rechallenge, and immunophenotyping.

## Visual Abstract

**Figure:**
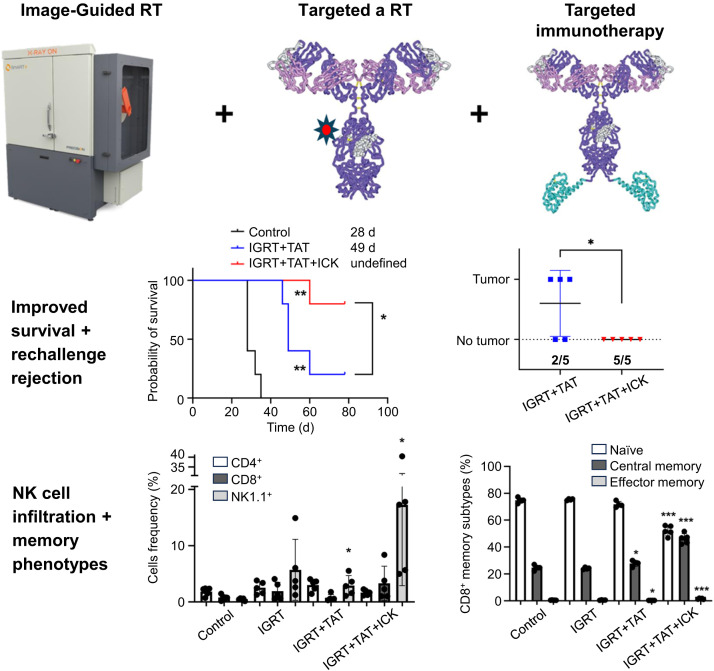

Even when immunotherapy is used as the first-line treatment for solid tumors, the response rate is low due to a refractory immune tumor microenvironment (TME) (1). Contributors to the immune TME include a poorly permeable tumor vasculature (2,3), inhibitory regulatory T cells (Tregs) (4) and myeloid cells (5), cancer-associated fibroblasts (6), and the absence of resident cytotoxic T cells (7). Before the administration of immunotherapy, conditioning the immune TME with low-dose targeted radiation therapy is an attractive approach (8). However, high-dose radiation should be avoided because it is likely to sterilize the immune TME, leading to accelerated wound healing or fibrosis (9). On the other hand, stimulation of an antitumor response is more likely to occur when low-dose radiation is used (10). Low-dose image-guided radiation therapy (IGRT) can be administered to small animals through the use of specialized equipment (11). Although this approach minimizes radiation to nontargeted organs, it cannot target spontaneous metastases that may occur before and after IGRT. Another approach is targeted radionuclide therapy, in which small molecules, peptides, or proteins, including antibodies, deliver a therapeutic radionuclide directly to the tumor (12). This approach targets both primary and metastatic tumors, as well as micrometastases but is limited by inherent systemic toxicity, as only a fraction of the administered agent reaches the tumor. Among the therapeutic radionuclides explored, α-particles are especially attractive because of their high linear-energy transfer and low tissue penetration, thus decreasing the chances of tumor cell DNA repair and limiting systemic toxicity (13). Since IGRT and targeted radionuclide therapy have distinct effects on the immune TME over time, there is a window during which immunotherapy can effectively take advantage of the proantitumor effects before wound repair reestablishes the refractory immune TME (10).

Since untargeted immunotherapy can result in unexpected side effects, including autoimmunity (14), tumor-targeted immunotherapy is preferred. Among immunotherapy approaches, bispecific antibody and chimeric antigen receptor T-cell therapy have shown promise but are not as effective in solid tumors where immune cell infiltration is low (15), and systemic cytokine release syndrome is a major side effect (16). The use of antibody–cytokine fusion proteins, called immunocytokines, is especially attractive (17), as immunocytokines can deliver cytokines such as interleukin 2 (IL-2) to the tumor activating the patients’ own cytotoxic T cells while reducing Treg numbers within the tumor and adjacent lymph nodes (18). Although immunocytokine therapy has shown success as a monotherapy, we have shown that it can be further improved by the addition of either IGRT (18) or targeted α-therapy (TAT) (19).

Given the inherent challenges of treating immunologically refractive tumors, we combined IGRT and TAT for the treatment of an immunocompetent solid tumor model expressing a human tumor antigen (20) before immunocytokine therapy, thus incorporating 3 types of targeted therapy.

## MATERIALS AND METHODS

### Cell Lines

Murine breast cancer E0771 and colon cancer MC38 cells stably transfected with carcinoembryonic antigen (CEA) were used, as previously described (18). Cell cultures were tested annually for the presence of mycoplasma.

### Animal Studies

Animal studies were performed in accordance with Institutional Animal Care and Use Committee Protocol 91017 and approved by the City of Hope Institutional Animal Care and Use Committee. Immunocompetent CEA transgenic mice on a C57BL/6J background were used, as previously described (20). Details of the experimental design are provided in the supplemental materials, available at http://jnm.snmjournals.org.

### PET Imaging Studies

Humanized anti-CEA antibody huM5A was conjugated with NHS-DOTA and radiolabeled with ^64^Cu, as previously described (19). PET imaging studies were performed in CEA transgenic mice bearing E0771/CEA tumors, using established methods (21).

### Leukocyte Analysis

Tissues were collected and analyzed by flow cytometry, as described previously (18). Single-cell suspensions were stained with different combinations of fluorochrome-coupled antibodies against CD3, CD4, CD8, CD11b, Ly6C, Ly6G, CD11c, F4/80, NK1.1, PD-1, CTLA-4, and Tim-3 (BioLegend). The expression of interferon gamma (IFNγ) and FoxP3 was studied using intracellular flow cytometry, as described previously (18).

### Statistical Analysis

Unpaired *t* test and 2-way ANOVA were used to determine *P* values. Survival analysis was performed using the Mantel–Cox log-rank test.

## RESULTS

### Effect of IGRT on Antibody Penetration into Tumors

**FIGURE 1.**
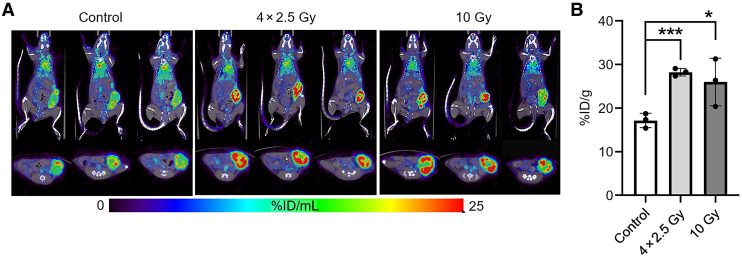
Effects of IGRT on antibody penetration into orthotopic breast tumors. (A) ^64^Cu-DOTA-anti-CEA antibody M5A (30 µg; 3.7 MBq) was administered 1 d after 0 or 10 Gy of IGRT or 4 doses of 2.5 Gy administered daily to CEA transgenic mice bearing established CEA transfected E0771 breast tumors. PET imaging was performed at 24 h. Upper row shows maximum-intensity-projection images; lower row shows stacked coronal tumor slices. (B) Mice were euthanized after PET imaging, tumors collected, and decay-corrected radioactivity was reported as percentage injected dose per gram of tissue (%ID/g) (*n* = 3 per group). *P < 0.05; ***P < 0.001.

A major drawback of antibody-targeted therapy is the poor penetration of antibodies into the TME (22). To determine if IGRT improved tumor penetration, we imaged CEA-positive (CEA+) tumors with radiolabeled anti-CEA antibody before and after IGRT using either a single dose of 10 Gy or a fractionated 10-Gy dose (4 doses of 2.5 Gy). Scattered radiolabeled antibody uptake was found in the untreated controls, while tumors treated with a single-dose of 10 Gy of IGRT showed an improved uptake, and those receiving the 4 fractionated doses of IGRT demonstrated a more uniform uptake (Fig. 1; Supplemental Table 1).

Thus, both single- and fractionated-dose IGRT improved antibody penetration, most likely due to changes in tumor vascular permeability, partly attributable to the reduction of high fluid pressure within the TME (23). Notably, both doses were nonsterilizing. Antibody penetration was more significant with fractionated-dose IGRT, potentially due to the delayed onset of antiinflammatory wound healing in the tumor, as the radiation dose was spread over time.

### Combination of IGRT and TAT in a Breast Cancer Model

Before performing experiments with 2 types of radiation therapy before immunotherapy, each of the radiation monotherapies were evaluated in 2 CEA+ tumor model systems to determine if IGRT plus TAT was more effective than either therapy alone. In the first study, the orthotopic breast tumor line E0771 transfected with CEA was used in CEA transgenic mice to mimic the situation in CEA+ human breast cancer (18).

**FIGURE 2.**
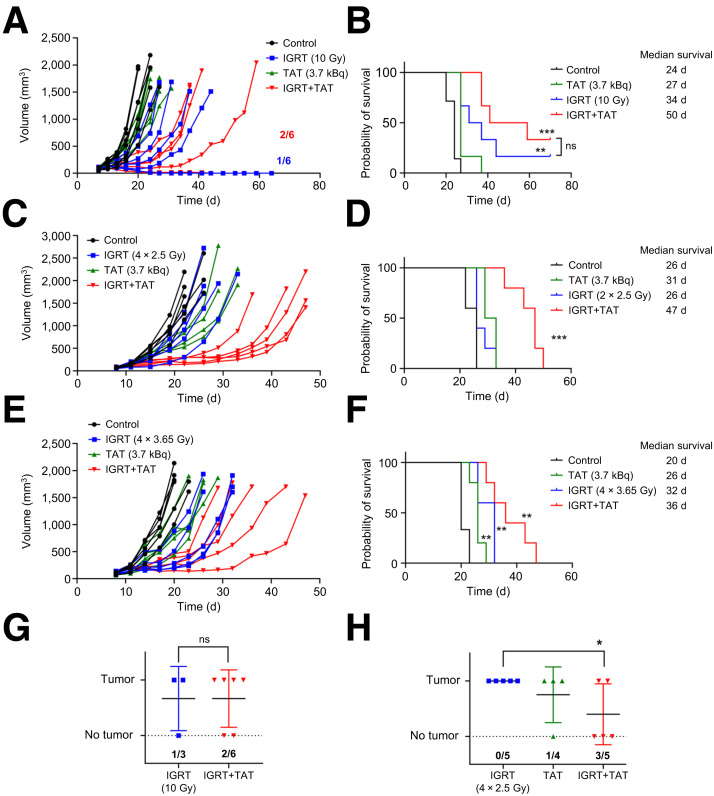
Comparisons of monotherapies with IGRT or TAT vs. combined IGRT plus TAT in immunocompetent breast cancer model. (A and B) CEA transgenic mice bearing established CEA transfected E0771 orthotopic breast tumors (*n* = 6) were treated with single dose of 3.7 kBq of ^225^Ac-DOTA-M5A (TAT) or single 10-Gy dose of IGRT or 10 Gy of IGRT followed by TAT 1 d later vs. untreated controls (*n* = 6 per group). Tumor growth (A) and probability of survival (B) measured over 50 d. (C and D) Similar study but with fractionated dose of IGRT (4 doses of 2.5 Gy over 4 d) or TAT only or combined IGRT plus TAT 1 d after completion of IGRT (*n* = 6 per group). (E and F) Similar study with 4 doses of 3.65-Gy IGRT or TAT 1 d after completion of IGRT or combined IGRT plus TAT (*n* = 5 per group). (G and H) Tumor rechallenge in opposite glands at end of studies shown in A and B and C and D. *P* values for treatment groups vs. untreated controls. *P < 0.05; **P < 0.01; ***P < 0.001.

In previous studies where we compared a single dose of 10 Gy to a fractionated-dose IGRT (4 doses of 2.5 Gy) (18), we realized that the cumulative fractionated dose was less than 10 Gy, calling into question the need for an equidose comparison. To determine whether a cumulative fractionated dose equal to 10 Gy was more effective than four 2.5-Gy doses, we studied the effects of four 3.65-Gy doses, using the formula *D*_new_ = 10 = *D*_old_ × 4^0.27^ (24). The results showed that a single 10-Gy dose of IGRT was more effective than either of the 2 fractionated doses in terms of survival curves (Fig. 2). In addition, 1 of 6 mice treated with a single dose of 10 Gy was cured (Fig. 2A). However, a single dose of 10-Gy IGRT plus low-dose TAT was more effective than a single dose of 10 Gy in terms of survival, including tumor cure in 2 of 6 mice, even though TAT alone was barely effective in delaying tumor growth compared with untreated controls (Figs. 2A and 2B). Of note, we selected 3.7 kBq for low-dose TAT on the basis of our previous study, which accounted for the high relative biologic effectiveness of α-emissions, including the emission of 4 α-particles, and the long half-life of ^225^Ac when combining TAT with immunotherapy (19). In terms of fractionated IGRT plus TAT, four 2.5-Gy doses of IGRT were slightly more effective than four 3.65-Gy doses of IGRT plus TAT (Figs. 2C–2F). In addition, we tested tumor rechallenge in the opposite flank in mice with the best responses against primary tumors to determine whether antitumor immunity was established for the single 10-Gy dose versus the fractionated doses (4 doses of 2.5-Gy IGRT) with and without TAT (Figs. 2G and 2H). The results indicated that the combination of 4 doses of 2.5-Gy IGRT plus TAT was the most effective, with 3 of 5 mice rejecting the tumor rechallenges (Fig. 2H).

### Combination of IGRT and TAT in a Colon Cancer Model

**FIGURE 3.**
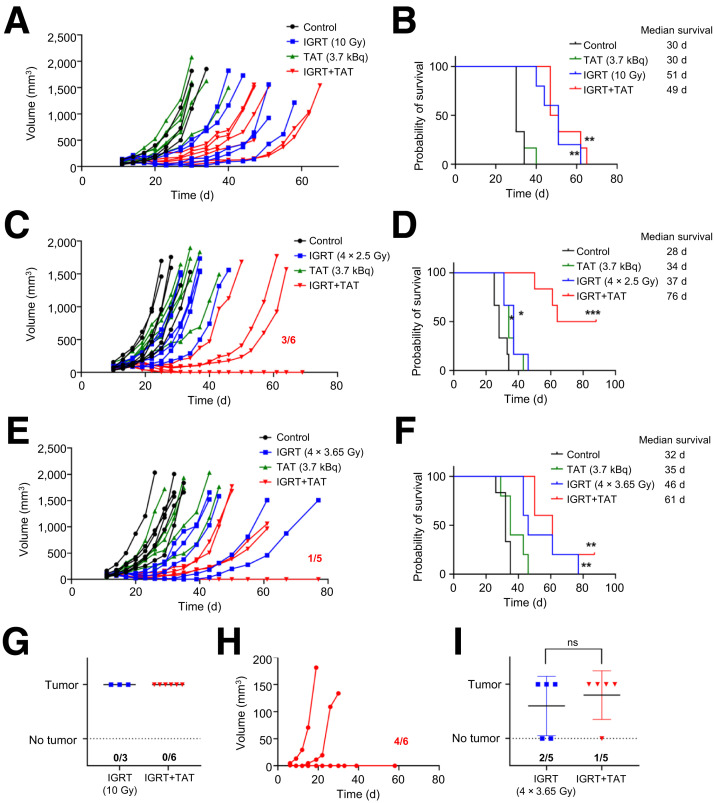
Comparisons of monotherapies with IGRT or TAT vs. combined IGRT plus TAT in colon cancer model. (A and B) CEA transgenic mice bearing established CEA transfected MC38 tumors (*n* = 6) were treated with single 3.7-kBq dose of ^225^Ac-DOTA-M5A (TAT) or single 10-Gy dose of IGRT followed by TAT vs. untreated controls (*n* = 6 per group). Tumor growth (C) and probability of survival (D) measured over 80 d. Similar study but with 4 doses of 2.5-Gy IGRT over 4 d or TAT only or 4 doses of 2.5-Gy IGRT plus TAT 1 d after completion of IGRT (*n* = 6 per group). (E and F) Similar study with 4 doses of 3.65-Gy IGRT or TAT 1 d after completion of IGRT or combined IGRT plus TAT (*n* = 5 per group). (G–I) Tumor rechallenge in opposite flanks at end of studies A and B, C and D, and E and F. *P* values for treatment groups vs. untreated controls. *P < 0.05; **P < 0.01; ***P < 0.001.

The analogous study was repeated in a second tumor model in which CEA was transfected into the murine colon carcinoma line MC38 grown in CEA transgenic mice (19). The colon cancer model revealed that the most effective combination therapy was four 2.5-Gy doses of IGRT plus low-dose (3.7 kBq) TAT. Importantly, a single 10-Gy dose of IGRT with or without TAT performed similarly (Figs. 3A and 3B), but there was a dramatic improvement with the addition of TAT to four 2.5-Gy doses of IGRT (Figs. 3C and 3D). Noteworthy, four 3.65-Gy doses of IGRT with or without TAT (Figs. 3E and 3F) performed no better that their monotherapy IGRT regimens, suggesting that lower-cumulative-dose IGRT worked better with low-dose TAT, a monotherapy that was ineffective by itself. Thus, the results indicated that the lower dose (delivered by four 2.5-Gy doses of IGRT) plus TAT was more effective in delaying tumor growth with increased survival than either the single 10-Gy dose or fractionated 10-Gy equidose IGRT plus TAT regimens. Similar to our findings with the breast tumor model, monotherapy with low-dose TAT was ineffective at reducing tumor growth, indicating that low-dose TAT was dramatically assisted by prior IGRT. When each of the treated groups was rechallenged with new tumors in the opposite flank, 4 of 6 were rejected when treated with four 2.5-Gy doses of IGRT plus TAT, none were rejected with the single 10-Gy dose plus TAT, and 1 of 5 were rejected with the fractionated equidose 10-Gy IGRT plus TAT (Figs. 3G–3I).

### Combination Therapy for Colon Cancer Tumors with Fractionated IGRT Plus TAT Followed by Immunotherapy

Comparing the results from the 2 CEA+ tumor models, we selected the colon cancer model for further treatment with immunotherapy. The colon cancer model allowed 50% mice to survive for a longer period (80 d), and this model benefitted most from the combination of IGRT plus targeted immunotherapy or TAT plus targeted immunotherapy in previous studies (18,19).

**FIGURE 4.**
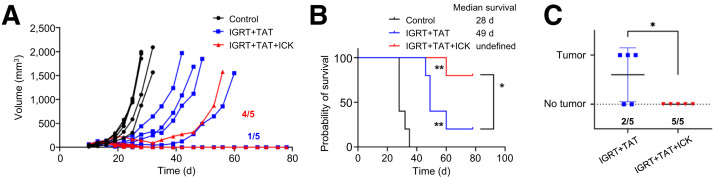
Combination IGRT/TAT/immunocytokine therapy in colon cancer model. (A and B) CEA transgenic mice bearing established CEA transfected MC38 tumors (*n* = 5) were treated with 4 doses of 2.5-Gy IGRT over 4 d followed by 3.7 kBq of ^225^Ac-DOTA-M5A TAT 1 day later, then 10 d after TAT with 1 mg/kg immunocytokine therapy daily for 4 days vs. IGRT plus TAT or untreated controls (*n* = 5 per group). Tumor growth (A) and probability of survival (B) measured over 80 d. (C) At 28 d after tumor injection, both treatment groups were rechallenged with tumors in opposite flanks and tumor regrowth recorded over 47 d. *P < 0.05; **P < 0.01; ICK = immunocytokine therapy.

In those studies, as well as the current study, we selected anti-CEA-IL-2 immunocytokine as the targeted immunotherapy. In the IGRT plus TAT treatment group, 1 of 5 tumors were eradicated, and 4 of 5 tumors in the group treated with IGRT plus TAT plus immunocytokine therapy (IGRT/TAT/immunocytokine therapy) (Fig. 4A). Survival was monitored for 78 days and represented with survival curves, showing significant survival improvement in the triple-therapy group (Fig. 4B). All treated mice received a second tumor injection in their opposite flanks at 28 d to assess the establishment of immune memory. No new tumors grew in the IGRT/TAT/immunocytokine therapy group, whereas 3 of 5 tumors regrew in the IGRT plus TAT group (Fig. 4C). We concluded that low-dose fractionated IGRT combined with low-dose TAT plus immunocytokine therapy was more effective than single-dose IGRT combined with TAT plus immunocytokine therapy in achieving tumor immunity, indicating that 2 types of combined targeted radiotherapy improved subsequent targeted immunotherapy and that fractionated IGRT outperformed single-dose IGRT in this approach.

### Early Immunophenotype Analysis with IGRT/TAT/Immunocytokine Therapy

The effect of various therapies and their combination on the tumors, tumor-draining lymph nodes (TDLNs), and the spleen must be evaluated within a few days after the start of therapy. By the end of the abovementioned studies, the tumors were absent or, if regrowing, reverted to the immune-resistant phenotype of untreated tumors. Therefore, we performed a study comparing tumors treated with IGRT monotherapy, IGRT plus TAT, and IGRT/TAT/immunocytokine therapy with untreated controls. Tumors, TDNLs, and spleens were harvested 2 d after each treatment was administered.

**FIGURE 5.**
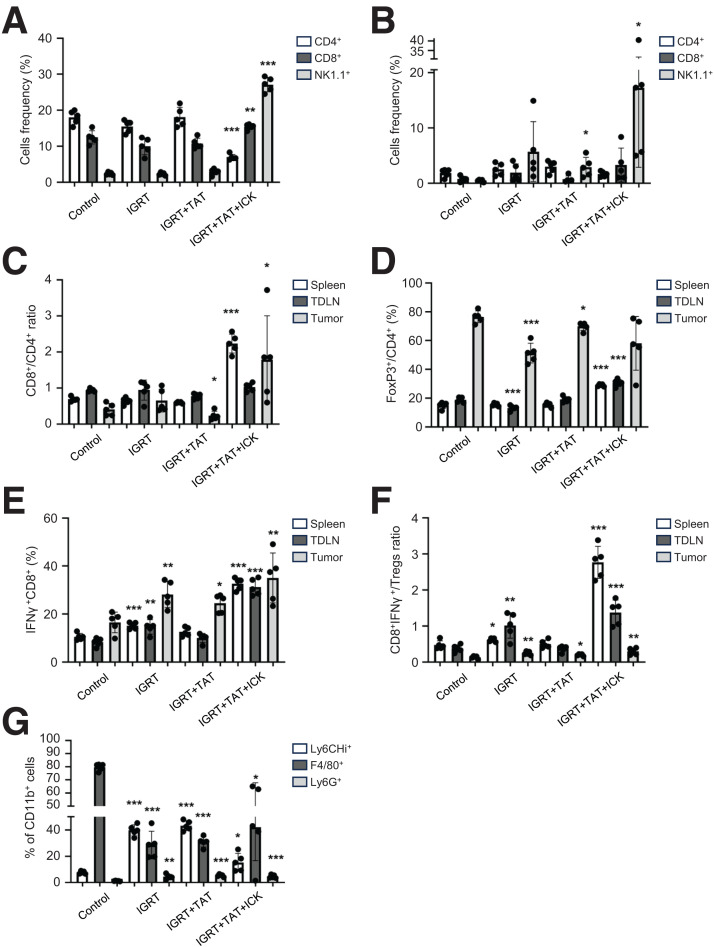
Selected immunophenotypes in CEA transgenic mice bearing CEA+ colon cancers after treatment with IGRT, IGRT plus TAT, or IGRT/TAT/immunocytokine therapy vs. untreated controls. (A and B) Frequency of T cells and NK cells in spleen (A) and tumor (B). CD8+/CD4+ T cell ratios (C) and percentage of CD4+ Tregs (D) in tumor, TDLN, and spleen. (E and F) Percentage of IFNγ+ CD8+ T cells (E) and their ratio to CD4+ Tregs in tumor, TDLN, and spleen (F). (G) Phenotypes of tumor myeloid cells (*n* = 5 per group). *P < 0.05; **P < 0.01; ***P < 0.001; ICK = immunocytokine therapy.

When 3 types of infiltrating immune cells in the spleen were analyzed for the 3 treatment groups and the untreated controls, significant changes were found only in the IGRT/TAT/immunocytokine therapy group, where the frequency of CD4-positive (CD4+) T cells decreased, CD8-positive (CD8+) T cells increased, and natural killer (NK) cells increased by almost 6-fold (Fig. 5A). This result highlights the profound effects of circulating immunocytokines on IL-2–responding cells (25). The same analysis showed a large increase in the number of NK cells in the tumors, but the significance of this finding was reduced by a varied tumor response (Fig. 5B).

Next, we analyzed the ratios of CD4+-to-CD8+ T cells versus the percentage of Tregs (FoxP3+/CD4+ T cells) in spleens, TDLNs, and tumors (Fig. 5C). This was important for 2 reasons. First, whereas IL-2 alone is known to increase the number of Tregs, the opposite is true for IL-2 in immunocytokines (18). Second, since the overall percentage of CD4+ T cells was reduced while the percentage of CD8+ T cells increased in the spleens of mice treated with IGRT/TAT/immunocytokine therapy, the reduction of the CD4+ Treg population was likely responsible for the decrease in the percentage of CD4+ T cells. Thus, the overall effect would result in more-effective CD8+-driven antitumor therapy, as previously reported (18). Indeed, this was the case for tumors that exhibited reductions in CD4+ Tregs when treated with IGRT/TAT/immunocytokine therapy (Fig. 5D). Interestingly, the decrease in tumor CD4+ Tregs was also seen with IGRT monotherapy and, to a lesser extent, with IGRT plus TAT. In any case, the high percentage of CD4+ Tregs in untreated controls is a likely driver of tumor resistance, and their reduction relative to CD8+ T cells is essential to a robust antitumor response.

Given the importance of infiltrating cytotoxic CD8+ T cells as measured by their production of IFNγ, we analyzed both their frequency and ratio to CD4+ Tregs (Figs. 5E and 5F). As expected, both the CD8+ IFNγ+ T-cell frequency and their ratio to CD4+ Tregs was highest in the IGRT/TAT/immunocytokine therapy group for the spleen, TDLNs, and tumors, with notable effects in the IGRT monotherapy group. The less-pronounced effect in the IGRT plus TAT group may be due to the chosen window for analysis, since the addition of TAT prolongs or complicates the therapy duration due to the toxic effects of the α-therapy on radiation-sensitive immune cells.

Since we were also interested in the myeloid component of the tumors, we analyzed subsets of CD11b+ cells: Ly6C^Hi^ (monocytes), Ly6G+ (granulocytes), and F4/80+ macrophages. The results showed a preponderance of macrophages (80%) in untreated controls that were significantly reduced and mostly replaced by monocytes in all 3 therapies (Fig. 5G). Strikingly, both IGRT monotherapy and IGRT plus TAT significantly increased the frequency of granulocytes, which remained high but reduced in number when compared with untreated controls for IGRT/TAT/immunocytokine therapy. Thus, the change in the TME as reflected by the reduction of macrophages and infiltration of granulocytes and monocytes is an important feature of these therapies. It is likely that, without these changes in the TME, antitumor immune infiltration and the positive effects of immunocytokine therapy would have been greatly diminished.

### Sources of Infiltrating CD4+ and CD8+ T Cells

**FIGURE 6.**
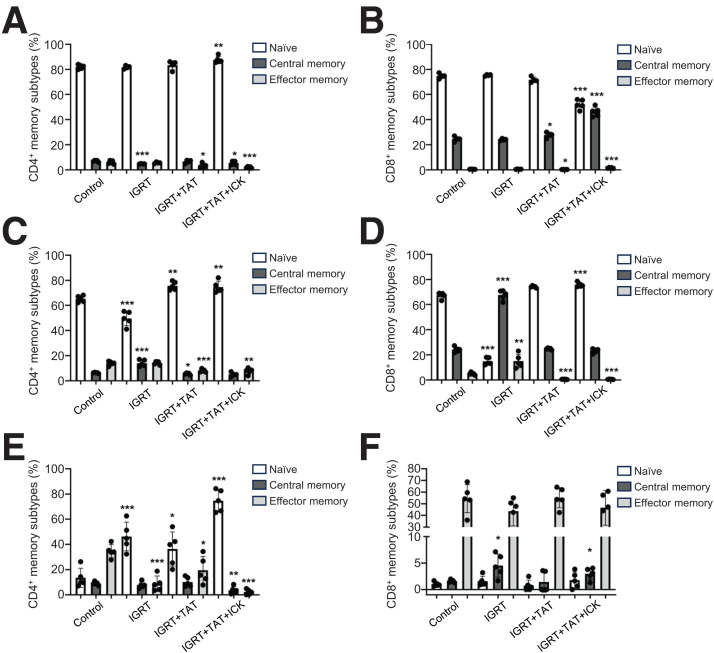
Origin of CD4+ and CD8+ T cells in CEA transgenic mice bearing CEA+ colon cancers after treatment with IGRT, IGRT plus TAT, or IGRT/TAT/immunocytokine therapy vs. untreated controls. (A–F) Frequency of CD4+ and CD8+ naïve (CD62L+CD44−), central (CD62L+CD44+), and effector (CD62L−CD44+) memory T cells in TDLN (A and B), spleen (C and D) and tumor (E and F) (*n* = 5 per group). *P < 0.05; **P < 0.01; ***P < 0.001; ICK = immunocytokine therapy.

Given that tumor-infiltrating CD4+ and CD8+ T cells may arise from naïve, central memory, or effector memory precursor cells (26), we analyzed CD4+ and CD8+ T cells from the spleen, TDLNs, and tumors of mice receiving the 3 types of therapy and compared the results with untreated controls (Fig. 6).

As expected, the majority of CD4+ T cells in the TDLNs were of the naïve phenotype, and the changes with therapy were minor in magnitude but were significant when compared with untreated controls (Fig. 6A). However, the CD8+ T-cell phenotypes of TDLNs changed from mostly naïve to an equal mix of naïve and central memory cells in the mice treated with IGRT/TAT/immunocytokine therapy (Fig. 6B).

For spleens, a more complex picture was seen for CD4+ T cells. Depending on the therapy, the number of naïve CD4+ T cells decreased with IGRT but increased with IGRT plus TAT or IGRT/TAT/immunocytokine therapy (Fig. 6C). The decrease in naïve CD4+ T cells with IGRT appears to be offset by an increase in central memory CD4+ T cells, perhaps an unappreciated feature of low-dose IGRT that requires further investigation. In terms of subsets of CD8+ T cells in spleens, the most dramatic change was seen with low-dose IGRT, where central memory CD8+ T cells increased almost 3-fold at the expense of naïve CD8+ T cells (Fig. 6D). Thus, changes in both CD4+ and CD8+ T cells were provocative for spleens with IGRT, emphasizing the importance of this modality in preparing the tumor for a productive antitumor response.

In contrast to the spleen and TDLNs, there was almost a complete lack of naïve CD4+ T cells in the control tumor, a situation that was reversed by all 3 types of therapy, with IGRT/TAT/immunocytokine therapy exhibiting the most dramatic increase (Fig. 6E). On the other hand, effector memory CD4+ T cells, whose number was strikingly low in the spleen and TDLNs in controls, comprised the major population of CD4+ T cells in the untreated tumors. In terms of the tumor CD8+ T-cell phenotypes, effector memory CD8+ T cells were the predominant CD8+ T cells (>50%) in the controls and 3 treatment groups and changed very little in the 2-d window analyzed, including a small but significant increase of the central memory subset with IGRT alone and IGRT/TAT/immunocytokine therapy (Fig. 6F). Given the few changes observed at the 2-d time point and the relative importance of cytotoxic CD8+ T cells for antitumor effects, it appeared that the key phenotypic events leading to their predominance had already occurred. However, since these effector memory CD8+ T cells are already present in the untreated controls, we concluded that they were ineffective in killing tumor cells. This can be explained by a requirement for CD4+ T helper cells to convert them to IFNγ-producing cells resulting in effective tumor therapy and reduction of Tregs.

## DISCUSSION

There is a growing consensus that the effectiveness of immunotherapy alone, though often ineffective for solid tumors, can be dramatically improved by the prior use of low-dose targeted radiation therapy (8,27–31). In this study, we found that imaging a tumor before and after IGRT provided insights into both the degree of antibody penetration and total uptake (Fig. 1; Supplemental Table 1), suggesting that this could be a general method for evaluating the effects of radiation on tumor vascular permeability and the subsequent uptake of therapeutic agents. In terms of dose of IGRT, a fractionated scheme was better than a single high dose, similar to that predicted by Demaria et al. (31), to increase immune infiltration. In terms of the TAT dose for immune infiltration, we previously found that, compared with 2 higher doses, the lowest dose (3.7 kBq) of ^225^Ac-DOTA-M5A increased tumor infiltration of CD8+ T cells (19). However, at this low dose, little to no tumor reduction was observed. Thus, in this study, low-dose TAT mainly provided an immunostimulatory effect. In terms of the best IGRT dose to prepare the tumor for low-dose TAT, 2 key findings emerged: fractionated IGRT performed better than a single 10-Gy dose and lower-dose fractionated IGRT performed better than the 10-Gy fractionated equidose. This result emphasizes that the 2 different types of radiation therapy (IGRT and TAT) have different biologic effects (32,33).

It is well known that targeted radionuclide therapy transforms immunologically cold tumors into immune-responsive tumors when combined with untargeted checkpoint blockade therapy (34). In one study, the addition of external beam radiotherapy with targeted radionuclide therapy allowed for the targeting of the TME, providing long-lasting antitumor responses (34). However, a detailed assessment of the immune effects was lacking in that study. In this study, we demonstrated that fractionated IGRT plus low-dose TAT before targeted immunotherapy is effective in overcoming a refractory immune TME with long-lasting antitumor effects. Further studies are required to optimize both the dose and timing of fractionated IGRT, as well as the minimum low dose for TAT that improves the immune response. Exploring all possible combinations of a triple-therapy approach is beyond the scope of this initial study.

Nonetheless, it appears that the goal of inducing complete inhibition of tumor growth, as well as tumor immunity, is possible. Moreover, immunophenotyping performed in a separate experiment 2 d after the completion of all 3 therapies showed major changes in the population of CD8+/IFNγ+ T cells versus CD4+/FoxP3+ Tregs, especially their ratios, and correlated well with therapy efficacy, similar to our previous studies (18,19). Interestingly, NK cells in the spleen and tumors showed a 6-fold increase in the triple-therapy group (Figs. 5A and 5B), making them the most numerous immune cells in the spleen and tumors (28 and 18%, respectively). Although the sole use of the NK1.1 marker did not exclude the potential role of NK T cells, it is well known that the early infiltration of NK cells into tumors correlates with a robust antitumor response (35,36). Thus, by percentages alone, we can conclude the NK response dominated the systemic and tumor compartments in the triple-therapy group at the 2-d time point.

In this study, we found that exposing tumors to radiation affects the composition of myeloid cells by reducing F4/80+ macrophages and increasing Ly6C^Hi^ monocytic cells (Fig. 5G), which could include myeloid-derived suppressive cells, known to infiltrate irradiated tumors and limit cytotoxic T cells (37).

In terms of tracking the origins of the antitumor immune cells, we found that a central memory subset of tumor-infiltrating CD8+ T cells provided superior antitumor immunity compared with effector memory CD8+ T cells, similar to the findings of a comparative study by Klebanoff et al. (38). Interestingly, Yang et al. (39) recently showed that tumor-associated monocytes correlated with the accumulation of CD8+ memory T cells in human cancers and selectively reprogram CD8+ T cells into central-memory-like T cells. That finding may be correlated with the tumor infiltration of radiation-induced Ly6C^Hi^ monocytes observed in our study. Overall, these analyses highlight the tumor-specific effects of immunocytokine-targeted IL-2, promoting the growth of CD25+ NK cells while reducing the number of CD25+ Tregs. This differential sensitivity of NK cells (stimulation) versus Tregs (reduction) is likely due to the Fc effector function of immunocytokines, similar to that observed for IL-2-Fc effects on Tregs (40). Future studies that examine later time-dependent immune population changes in the spleen, TDLNs, and tumors may reveal more details about the mechanism of the shift from immune resistance to immune killing of tumor cells.

## CONCLUSION

Using 2 syngeneic tumor models, we showed that low-dose fractionated IGRT combined with low-dose TAT showed the highest tumor growth inhibition and immune memory against tumor rechallenge. Moreover, the combination of these radiation therapies, followed with tumor-targeting immunocytokine therapy, had the best therapeutic effects, as evidenced by tumor cure, rejection of tumor rechallenge, and immunophenotyping indicating the dominant effector responses exerted by cytotoxic NK and CD8+ T cells. Based on these preliminary findings, further studies to explore the doses and timing of IGRT, TAT, and immunocytokine combination therapies are warranted.

## DISCLOSURE

This research was supported by NIH grant CA283603 and cancer center support grant P30CA033572. No other potential conflict of interest relevant to this article was reported.

## Notes

KEY POINTS **QUESTION:** Can IGRT and TAT improve subsequent antibody-targeted immunotherapy in an immunocompetent model? **PERTINENT FINDINGS:** Three types of IGRT (single dose, fractionated equidose, and fractionated low dose) were combined with low-dose TAT to determine the best combination of IGRT plus TAT in 2 CEA+ tumor models prior to selecting the best combination of IGRT plus TAT for inclusion with immunocytokine therapy. Low-dose fractionated IGRT plus TAT performed best and was further improved with immunocytokine therapy, including significant differences in immune cell populations in tumors, spleens, and lymph nodes analyzed 2 d after the completion of combined therapies. **IMPLICATIONS FOR PATIENT CARE:** Imaging a tumor before and after IGRT provided insights into both the degree of antibody penetration and total uptake, suggesting that this could be a general method for evaluating the effects of radiation on tumor vascular permeability and the subsequent uptake of therapeutic agents.
